# Secret heroes of the sea: brown macroalgae and their bioactive powers—a narrative review

**DOI:** 10.3389/fnut.2026.1766041

**Published:** 2026-02-23

**Authors:** Basak Can, Nevin Sanlier

**Affiliations:** 1 Department of Nutrition and Dietetics, Faculty of Health Sciences, Istanbul Beykent University, Istanbul, Türkiye; 2 Department of Nutrition and Dietetics, Faculty of Health Sciences, Ankara Medipol University, Ankara, Türkiye

**Keywords:** bioactive compounds, brown macroalgae, functional foods, marine bioactives, phytochemicals

## Abstract

Macroalgae have been used for nutritional and medicinal purposes in many cultures throughout history and they are an important part of traditional diets, especially in Asian countries. This narrative review provides an integrative overview of the effects of bioactive compounds present in brown macroalgae (Phaeophyceae) on nutrition and health. Brown macroalgae are rich in various bioactive compounds such as fucoxanthin, phlorotannin, fucoidan, alginate, and laminarin. These bioactive compounds have antioxidant, anti-inflammatory, antidiabetic, anticancer, and antihypertensive effects and may also exhibit immunoregulating or neuroprotective properties. Macroalgae contain high amounts of protein and polyunsaturated fatty acids, dietary fiber, vitamins, and minerals. Their nutrient contents vary depending on factors such as species, environmental conditions, and harvest time. Pigment and polyphenol derivatives, especially fucoxanthin and phlorotannins, have protective effects against chronic diseases associated with oxidative stress by reducing the effects of free radicals. However, there are very few studies on the bioavailability and mechanisms of the nutrients, phenolics, and flavonoids in macroalgae. Since the metabolic transformations of these metabolites in humans are overlooked, their effects on health are also unclear. More *in vivo* and clinical studies are needed on the potential use of brown macroalgae in the field of health. Overall, the findings summarized in this narrative review suggest that brown macroalgae represent promising, sustainable, and natural sources of bioactive compounds for future nutritional and health-related applications.

## 1 Introduction

Algae have long played an important role in human nutrition and traditional medicine, with archaeological evidence indicating their use as early as 14,000 years ago (1). They represent a highly diverse group of photosynthetic organisms adapted to a wide range of aquatic and semi-aquatic environments (2, 3). Based on morphological organization, algae are broadly classified into microalgae and macroalgae, with the latter comprising large, multicellular forms (4).

Among macroalgae, brown macroalgae have attracted particular scientific interest due to their rich content of structurally unique polysaccharides, polyphenols, and other bioactive compounds (5, 6). Previous studies have reported a range of biological activities associated with algal-derived compounds, including antioxidant, anti-inflammatory, antiviral, and metabolic regulatory effects (7–11). However, much of the existing evidence is derived from *in vitro* and experimental models, which limits the translation of these findings to human health applications.

In this context, the present review aims to provide an integrative overview of the nutritional composition and bioactive components of brown macroalgae and to critically discuss their potential health effects. Particular emphasis is placed on evaluating current evidence, identifying knowledge gaps, and outlining future research directions necessary for the development of evidence-based functional foods and nutraceutical applications.

In this review, the term “brown macroalgae” is used consistently to refer to members of the class Phaeophyceae, unless taxonomic context requires otherwise.

## 2 Methods

A literature search was conducted to identify relevant studies published between 2000 and 2025, with particular emphasis on recent experimental, preclinical, and human research. The search was performed using the PubMed, ScienceDirect, Web of Science, and Google Scholar databases. Keywords related to brown macroalgae and their health effects were combined using Boolean operators and included terms such as macroalgae, brown macroalgae, bioactive compounds, nutritional composition, polyphenols, polysaccharides, phlorotannins, fucoxanthin, diterpenes, fucoidan, alginate, human health, metabolism, antiviral, antidiabetic, antihyperlipidemic, anti-inflammatory, neuroprotective, antihypertensive, anticancer, and antioxidant. In addition, reference lists of relevant articles were manually screened to identify additional eligible studies.

All retrieved records were initially screened based on titles and abstracts to assess their relevance to the scope of this narrative review. Articles considered potentially relevant were subsequently evaluated through full-text reading. Studies focusing on brown macroalgae, their nutritional composition, bioactive compounds, and health-related effects were included, whereas publications with limited relevance to the aims of the review were excluded. Laboratory-based, cell-based, animal and human studies, as well as food chemistry studies, were taken into consideration.

Searches were limited to Turkish and English-language publications. No restrictions were applied regarding study design in order to capture a broad range of evidence relevant to the objectives of this narrative review. Duplicate records retrieved from multiple databases were identified and removed prior to screening. The findings from original research articles, as well as relevant reviews, were synthesized to provide an integrative overview of the current literature. As this work represents a narrative review, no formal quality assessment or risk-of-bias analysis was conducted. For transparency, the literature search and study selection process is summarized in Supplementary Figure 1.

## 3 Taxonomy of macroalgae

Macroalgae typically inhabit oceanic environments and are visible to the naked eye. They are distributed across various marine habitats with certain species exhibiting specific ecological preferences (12). The ecological roles and biogeographical distributions of macroalgae are significant factors in their taxonomy, as they may influence species diversity and evolutionary history (12, 13).

The number of extant macroalgae species remains a subject of debate; however, it is widely suggested that several thousand species exist. Macroalgae are macroscopic, eukaryotic, and photosynthetic marine organisms widely recognized for their ecological and nutritional relevance (12, 14). Although algae have been traditionally consumed in certain cultures, their integration into modern dietary practices varies considerably across regions. In recent years, interest in macroalgae has increased, particularly within plant-based dietary patterns, largely due to their nutritional potential (15).

Macroalgae are generally composed of a thallus, which typically consists of a leaf-like blade and a root-like holdfast. They exhibit considerable morphological diversity, ranging from simple crust-like forms to more complex foliose and filamentous structures (16). Based on pigmentation and phylogenetic characteristics, macroalgae are broadly classified into three main groups: Chlorophyta (green algae), Rhodophyta (red algae), and Phaeophyceae (brown macroalgae) (13, 17). Among these, brown macroalgae (Phaeophyceae) represent the most widely consumed group and are characterized by pigments such as chlorophyll a and c, β-carotene, and xanthophylls (18).

## 4 Brown macroalgae

Brown macroalgae are classified as photosynthetic organisms, considered as heterokonts or stramenopiles and belonging to the class Phaeophyceae. Their characteristic brown color arises from the presence of the carotenoid fucoxanthin and various phaeophycean tannins, in addition to green pigments such as chlorophyll a and c located in their chloroplasts. Species growing in deeper waters are capable of absorbing light at different wavelengths due to these pigments, enabling them to produce nutrients through photosynthesis. Brown macroalgae thrive predominantly in temperate to subpolar regions, where they exhibit the greatest diversity in both species richness and morphological forms (16). Brown macroalgae are the largest macroalgae, typically inhabiting shallow waters or rocky coastal zones, and are characterized by highly variable morphological structures and flexible thalli that enable them to withstand constant wave action. Some species can reach lengths of 35 to 45 meters. The most common genera include *Ascophyllum*, *Laminaria*, *Saccharina*, *Macrocystis*, *Nereocystis*, and *Sargassum* (19).

Brown macroalgae are rich in nutrients and secondary metabolites that exhibit a wide range of biological activities. The levels of these compounds vary depending on the species, harvest season, geographical origin, and environmental conditions (16). The production of the bioactive compounds they contain is affected by environmental factors such as hydrodynamics, temperature, light, and salinity (20). Furthermore, the ability of brown macroalgae to tolerate metal pollution through mechanisms driven by metallothioneins and phytochelatins reflects their ecological resilience (21).

Algae are rich in various essential nutritional components including protein, vitamins, minerals, and polysaccharides with unique structures. In particular, brown macroalgae contain many bioactive compounds such as phlorotannin, fucoxanthin, diterpene, fucoidan, and alginate, which have been scientifically proven to have positive effects on health (18). The bioactive compounds in brown macroalgae are particularly notable for their health benefits as they possess anti-inflammatory properties (22) and play roles in dyslipidemia control (23), obesity reduction (24), diabetes management (25), immunomodulation (26), and hypertension regulation (27), among others. In addition, they may also contribute to the prevention of some types of cancer (28, 29). Polysaccharides, and especially alginate, fucoidan, and laminarin, have been shown to have antiviral, antimicrobial, and antitumoral activities as confirmed by various *in vitro* and *in vivo* studies (30). Similarly, phlorotannins specific to brown macroalgae have demonstrated antioxidant, anti-inflammatory, and anticancer activities (31, 32).

## 5 Nutritional composition of brown macroalgae

Brown macroalgae, which are rich in protein, vitamins, minerals, structurally unique polysaccharides, and bioactive compounds such as phlorotannin, fucoxanthin, fucoidan, and alginate, are being evaluated in studies on functional foods and the development of therapeutic agents for their positive effects on health. Although their nutrient compositions vary depending on the species, environmental conditions, and geographical location, they generally contain significant levels of essential nutrients and bioactive substances. In the food industry, they stand out with their high vitamin, mineral, and dietary fiber contents. In addition, polysaccharide derivatives are used as tissue regulators and preservatives in various products (33, 34).

### 5.1 Carbohydrates

Brown macroalgae are rich in carbohydrates, mainly in the form of polysaccharides such as alginate, fucoidan, and laminarin. These compounds not only contain abundant energy but also have prebiotic properties that support intestinal health. Studies have reported that the carbohydrate content in brown macroalgae varies between 45 and 67.09% in dry weight and that they are important sources of dietary fiber (24, 35, 36).

Fucoidan and alginate are the primary polysaccharides found in brown macroalgae. In addition to these compounds, they also contain functional polysaccharides such as laminarin, a β-(1 → 3)-glucan, which can account for approximately 35% of their dry weight (16, 37, 38). Laminarins, also referred to as laminarans or leucosins, belong to the glucan family and serve as storage metabolites in brown macroalgae. These compounds are found in high concentrations in the blades of macroalgae belonging to the genera *Laminaria* and *Saccharina*, while they are present in lower amounts in species such as *Ascophyllum*, *Fucus*, and *Undaria*. Laminarins are polysaccharides of relatively low molecular weight composed of β-(1 → 3)-linked glucose monomers, characterized by high sugar contents and low levels of uronic acids (33). No traces of this compound were found in the feces of Wistar rats fed laminarin, suggesting that laminarins are significantly fermented in the digestive tract and metabolized by the intestinal microflora (39). Oligosaccharides derived from brown macroalgae exhibit bioactive properties differing from those of the original polysaccharides they are derived from. Alginate oligosaccharides are particularly used as antioxidant agents in the food industry due to their free radical scavenging capacity. In addition, the ability of these oligosaccharides to promote phagocytosis in microglial cells suggests that they may have immunomodulatory effects on the central nervous system. It is suggested that thanks to these properties, they can be evaluated as nutraceutical agents for preventing or slowing down neurodegenerative disorders such as Alzheimer’s disease by reducing neuroinflammation (40).

### 5.2 Protein

With increasing pressure on current agricultural and food production systems to meet the rising global demand for nutrients, algae have recently emerged as a significant focus of research and interest due to their protein quality and yield (41). The protein and amino acid profiles of algae are generally comparable to those of beef; however, they are typically consumed in much smaller quantities (42). The protein contents of macroalgae show significant differences among species. *Sargassum marginatum* is reported to contain approximately 118.0 mg/g protein on a dry weight basis, while this value has been found to reach up to 180.0 mg/g in *Chaetomorpha linum*, a green algae species. These algal proteins offer rich nutritional profiles, especially in essential amino acids for human nutrition (43–45). The presence of essential and non-essential amino acids together further increases the nutritional value of these species. Some brown macroalgae species can have total amino acid contents of up to 24.07 grams per 100 grams of algae (46–48).

The digestibility of algal protein affects the nutritional value of the algae, while protein-polysaccharide interactions reduce digestive efficiency (42). Amino acid profiles can vary between species and these differences affect flavor profiles as well as nutritional properties. Various algae are deficient in tryptophan and lysine, while methionine, cysteine, and lysine deficiencies may particularly occur in brown macroalgae. In contrast, aspartic acid and glutamic acid are prominent in algae and contribute to their distinctive umami flavor (47, 48). These amino acids constitute a significant portion of the total amino acids in species such as *Fucus* and *Ulva* (4). The structure of the cell walls of brown macroalgae results in proteins being embedded within the polysaccharide matrix, which creates a barrier to protein extraction (49). In addition, phenolic compounds such as phlorotannins, which are only found in brown macroalgae, cause proteins to form complex bonds with phenolic compounds in the algal matrix, reducing protein extraction efficiency. These factors make it difficult to obtain proteins from brown microalgae (50).

### 5.3 Lipids

Brown macroalgae generally have low lipid contents, typically ranging from 0.25 to 5.21% of dry weight, with neutral lipids and glycolipids being the most common. The proportion of essential fatty acids found in algae is higher than that in terrestrial plants, and algae can synthesize long-chain polyunsaturated fatty acids (PUFAs) (51). Therefore, fatty acids such as eicosapentaenoic acid (EPA) and docosahexaenoic acid (DHA), which belong to the ω-3 fatty acid family, are found in significant amounts in algae (15). ω-3 and ω-6 fatty acids are abundant in brown macroalgae, ranging from 34 to 74% of the total lipid content. Monounsaturated fatty acids are generally oleic acid (C18:1), accounting for 21% of the total lipids in brown macroalgae (52). Although the lipid contents of brown macroalgae are generally low, these organisms have profiles rich in PUFAs (4). In addition, brown macroalgae have the potential to support cardiovascular and nervous system health thanks to their various lipid components, including various other saturated and unsaturated fatty acids (51). Sterols, especially fucosterol, are found in lipid membranes. Brown macroalgae species also contain a wide variety of polar phenols and phenol derivatives such as phlorotannins (16).

### 5.4 Dietary fiber

Dietary fiber constitutes a group of edible carbohydrate polymers that are resistant to digestive enzymes and cannot be absorbed in the small intestine. Thanks to those properties, fiber interacts directly with the intestinal microbiota. Intestinal microbes ferment the fiber and help produce beneficial metabolites such as short-chain fatty acids (SCFAs) (53). Sulfated polysaccharides are considered to be a characteristic group of macroalgae derived dietary fibers that are rarely found in terrestrial plants. Sulfated polysaccharides consist of a group of complex carbohydrate macromolecules with varying proportions of sulfate groups (54). Besides macroalgae, marine animals such as sea cucumbers and marine microorganisms also possess sulfated polysaccharides, but their abundance and diversity are less compared to macroalgae derived fiber, which is presumed to be directly related to major biological activities (55).

Soluble fiber, generally found in macroalgae, can constitute 52–85% of the total fiber content commonly found in green, red, and brown macroalgae and can be fermented to SCFAs that play critical roles in energy homeostasis and immune function (56). Typical soluble macroalgae derived fiber include the alginate, laminarin, and fucoidan found in brown macroalgae; the carrageenan, agar, and agarose found in red macroalgae; and the ulvan found in green macroalgae. Insoluble fiber is generally composed of compounds such as cellulose, lignin, hemicellulose, and starch and it usually has limited bioactive activities (35).

Fucoidan, found in brown macroalgae, constitutes a complex group of polysaccharides composed of sulfated L-fucopyranose residues at the O-2 and/or O-3 positions and branched chains of L-fucose 4-sulfate linked via α-(1 → 2) glycosidic bonds with a C3 ester group. It also contains trace amounts of xylose, galactose, mannose, and uronic acids (57). In addition to its structural complexity, fucoidan has attracted attention due to its various beneficial health effects. In one study, fucoidans derived from brown macroalgae such as *Laminaria japonica* and *Ascophyllum nodosum* were shown to reduce body weight, fasting blood glucose, hepatic steatosis, and systemic inflammation in mice with metabolic syndrome induced by a high-fat diet (58). Moreover, the antidyslipidemic effects of fucoidans are presumed to be associated with bile acid metabolism and cholesterol transport (56), while the anti-metabolic syndrome effects of fucoidan are thought to be associated with the alleviation of excessive oxidative stress and inflammation. In one study, fucoidan derived from *Fucus vesiculosus* significantly reduced the levels of reactive oxygen species (ROS) in HepG2 cells by attenuating sodium palmitate-induced insulin resistance through modulation of the JNK and Akt signaling pathways in a ROS-dependent manner (59).

Studies investigating the macronutrient and fiber contents of brown macroalgae are summarized in Supplementary Table 1.

### 5.5 Vitamins and minerals

Brown macroalgae contain significant amounts of various vitamins and minerals (43). They are rich in basic minerals such as calcium, magnesium, potassium, iodine, and iron, which are necessary for human health, but also have the potential to accumulate toxic elements from the environment (60).

The vitamin and mineral contents of macroalgae vary significantly depending on factors such as the species, the season, and the habitat. In addition, macroalgae groups differ significantly among themselves in terms of mineral absorption capacity. These variations are directly associated with environmental factors such as the physiochemical properties of the environment of the algae and their unique biological structures (42, 61). Depending on the type of algae and their origins, some species may contain particularly high concentrations of certain minerals. For example, *Pyropia yezoensis* (red algae), *Sargassum fusiformis* (brown macroalgae), and *Ulva intestinalis* (green algae) have iron levels in the range of 9.12–54.4 mg/100 g and zinc contents in the range of 0.82–5.24 mg/100 g, while other red, brown, and green algae have relatively high levels of calcium, sodium, and potassium (35). These minerals are particularly prevalent in brown macroalgae. In some species of brown macroalgae, the calcium contents may exceed 14,000 ppm. These minerals play critical roles in basic physiological processes such as the protection of bone health, regulation of blood pressure, and oxygen transport in the body (36). Other basic minerals such as magnesium, phosphorus, and zinc are also significant (45). Two key features distinguish the mineral profiles of these macroalgae from those of terrestrial plants: low sodium-to-potassium (Na/K) ratios and high iodine contents. Low Na/K ratios are known to be an important factor in the maintenance of cardiovascular health (62). The Na/K ratios of brown macroalgae have been reported to range between 0.3 and 1.5. In particular, species of *Laminaria* exhibit ratios between 0.3 and 0.4, which is significantly lower than those found in various common foods such as Cheddar cheese (8.7), olives (43.6), or sausages (4.9) (33). Iodine contents vary among edible brown algal species. These levels can be reduced through water-based processing methods performed at low temperatures, such as the washing, soaking, and rehydration of dried algae. For adults, the recommended upper intake level (UL) of iodine is 600–1,100 μg per day, which corresponds to the consumption of approximately 0.2 to 11 g of processed dried brown macroalgae. This amount varies depending on the algal species and the processing methods applied. Therefore, safe consumption levels of brown macroalgae can be said to depend on the harvesting location, processing conditions, certain properties of the algae, and the homogeneity of the raw materials (63). The iodine concentrations of *Laminaria digitata*, *A. nodosum*, *Himanthalia elongata*, and *Undaria pinnatifida* were, respectively, reported as 70, 18.2, 10.7, and 3.9 mg/100 g weight (33).

### 5.6 Bioactive compounds

Brown macroalgae attract attention with their various bioactive components in addition to their rich nutrient contents. However, the nutritional compositions of these organisms vary significantly depending on factors such as the species, seasonal changes, geographical distributions, and environmental conditions (34). Although these algal species are generally considered safe, regular monitoring of the possible accumulation of environmental pollutants such as heavy metals is of great importance for their safe consumption (34).

Algae contain bioactive compounds such as carotenoids and polyphenols with high antioxidant capacity. The natural pigments of algae have been investigated for various bioactivities including antioxidant, anticancer, and anti-inflammatory activities. Fucoxanthin, one of these natural pigments, is a xanthophyll carotenoid that structurally belongs to the tetraterpenoid class and attracts attention with the allenic bond and 5,6-monoepoxide ring in its structure (15, 64). Fucoxanthin contributes up to 70% of the total carotenoid contents of algae and it is the pigment that gives brown macroalgae their color (53). The levels of this pigment vary significantly among different species and may also fluctuate widely within the same species according to external factors. For instance, its levels have been reported as 171 mg/kg in *Fucus spiralis*, 224 mg/kg in *Fucus distichus*, 364 mg/kg in *Fucus evanescens*, 172–660 mg/kg in *A. nodosum*, and 178–468 mg/kg in *Laminaria* species (39). Fucoxanthin isolated from macroalgae has been reported to inhibit the differentiation of 3 T3-L1 preadipocytes into adipocytes by downregulating PPARγ expression (65). Additionally, a fucoxanthin-based diet incorporating *U. pinnatifida* was shown to induce the expression of uncoupling protein 1 (UCP1) in the white adipose tissue of obese mice (66). When fucoxanthin supplementation was provided to rats fed a high-fat diet, it caused a decrease in the mRNA expression of important enzymes associated with lipid metabolism. These enzymes included fatty acid synthase, acyl-CoA cholesterol acyltransferase, hepatic acetyl-CoA carboxylase, glucose-6-phosphate dehydrogenase, hydroxy-3-methylglutaryl coenzyme A, and SREBP-1C (66, 67). According to all these studies, fucoxanthin obtained from different algal species has antioxidant, weight management (68), anticancer, anti-inflammatory, antiobesity (69), neuroprotective (70), and antiosteoporosis (15) effects.

Polyphenols are powerful antioxidants and brown macroalgae produce these compounds to protect against external conditions such as stress and herbivores. The polyphenols of these algal species range from phenolic acids to flavonoids and from halogenated phenolics to phlorotannins (16). Phlorotannins are polyphenolic tannins commonly found in brown macroalgae and they are divided into six categories based on their chemical structures: (1) phlorethols, which contain aryl-aryl bonds; (2) fuhalols, characterized by aryl-ether linkages; (3) eckols, containing dibenzo-1,4-dioxin linkages; (4) fucophlorethols, possessing ether or phenyl linkages; (5) carmalols, which feature a dibenzodioxin unit; and (6) fucols, structures with ortho- or para-positioned ether bridges containing an additional hydroxyl group. The chemical diversity and biological functions of phlorotannins are better understood in light of these categories, and the antioxidant, anti-inflammatory, and antitumor properties of these polyphenolic compounds make them extremely valuable for therapeutic applications (53, 71).

Meroditerpenoids are derived in part from terpenoids and are characterized by having a polyprenyl chain attached to a hydroquinone ring moiety. These compounds are especially common in brown macroalgae belonging to the family *Sargassaceae* of the class Phaeophyceae. The meroditerpenoid group includes plastoquinones, chromanols, and chromenes. The biosynthesis of terpenoid precursors occurs via two main biosynthetic pathways, the MVA pathway and the MEP pathway (53).

## 6 Health effects of brown macroalgae

Brown macroalgae have emerged as a significant source of bioactive compounds with diverse pharmacological properties, attracting growing scientific interest for their potential roles in promoting human health and preventing chronic diseases. For example, brown macroalgae such as *Padina australis* contain flavonoids, alkaloids, saponins, and steroids with significant antioxidant activity as reflected by an IC_50_ value of 102.590 μg/mL, indicating the potential of these algae in neutralizing free radicals (72, 73). Supercritical extracts of brown macroalgae such as *Saccharina japonica* and *A. nodosum* are rich in carotenoids, phenolic compounds, and mannitol, which contribute to their high antiradical and superoxide radical scavenging activities (74, 75). Polysaccharides such as phlorotannins and fucoidan obtained from brown macroalgae also have antioxidant potential that helps alleviate oxidative stress and activate oxidative defense pathways (76).

### 6.1 Antioxidant effects

#### 6.1.1 Preclinical evidence (*in vitro* and animal studies)

The antioxidant properties of brown macroalgae have been associated with a variety of health benefits, including antidiabetic, anticoagulant, antiproliferative, antimicrobial, anti-inflammatory, and anticancer effects (77). Oxidative stress is caused by imbalances between the production and neutralization of free radicals, which can lead to various degenerative diseases. Some compounds isolated from marine macroalgae have been found to exhibit significant antioxidant activity, with bromophenols, phlorotannins, and flavonoids particularly standing out (78). It is also known that fiber obtained from macroalgae may exhibit strong antioxidant properties. A study of the Vero cell line showed that *Hizikia fusiforme* fucoidans increased the expression of endogenous antioxidant enzymes by increasing Nrf2 levels in cells treated with H₂O₂ (79). Phenolic compounds are classified as simple phenols or polyphenols according to the number of phenol units in their molecules. Bromophenols are marine-derived secondary metabolites that contain one or more bromine atoms in their phenol groups and have been isolated from various marine organisms, including red, brown, and green algae, as well as ascidians and sponges (80).

Phenolic-rich extracts from edible brown macroalgal species of the genera *Alaria* and *Ascophyllum* have been shown to inhibit the progression of colon cancer at low doses, while *Ascophyllum* extract can potently suppress α-amylase and α-glucosidase enzymes at micromolar levels (35). Fucosterol, a sterol isolated from brown macroalgae, has also been reported to exhibit antioxidant activity in hepatic cells by increasing glutathione levels and reducing ROS production, thereby exerting hepatoprotective effects against oxidative stress-induced liver damage, including reductions in ALT and AST levels (80, 81).

Carotenoids synthesized by algae exhibit strong antioxidant activity and play an important role in protecting algae against photo-oxidative stress through singlet oxygen quenching and free radical scavenging mechanisms (80). Brown macroalgae such as *U. pinnatifida*, *Costaria costata*, *S. japonica*, and *A. nodosum* are rich in phenolic compounds and carotenoids, which contribute to their strong antiradical activity, hydroxyl ion binding, and superoxide radical scavenging capacities (82).

Compounds derived from brown macroalgae, including sulfated polysaccharides and polyphenols, offer therapeutic potential for the management of metabolic diseases by regulating signaling pathways associated with oxidative stress (83). Fucoxanthin, commonly found in brown macroalgae, is particularly notable for its antioxidant and antimelanogenic activities and has attracted interest as a bioactive ingredient for cosmetic and pharmaceutical applications (64). Its antioxidant effects are mainly attributed to singlet oxygen quenching, free radical scavenging, and inhibition of lipid peroxidation. In addition, fucoxanthin has been reported to suppress vascular endothelial growth factor overexpression, improve phagocytic function, and protect retinal cells from photo-induced oxidative damage (81). Various extracts obtained from brown macroalgae exhibit protective effects in different biological systems due to their antioxidant capacities. For example, *Sargassum binderi* extract demonstrated nephroprotective effects in experimental models by reducing oxidative stress-induced kidney damage (84). Natural antioxidants derived from brown macroalgae are therefore considered promising alternatives to synthetic antioxidants, which may be associated with adverse effects (85).

In addition, several extraction techniques, including ultrasound-assisted extraction, vortex-assisted extraction, and high-pressure-assisted extraction, have been employed to optimize the antioxidant yield of brown macroalgae. The extraction method selected can significantly influence total phenolic content and antioxidant capacity, and antioxidant activity has been reported to be retained even after air-drying processes in certain species, such as *Lobophora dagamae* (86, 87).

#### 6.1.2 Clinical evidence and translational considerations

However, it should be noted that most evidence supporting the antioxidant effects of brown macroalgae is derived from *in vitro* and animal studies, whereas human data remain limited. Recent systematic reviews and narrative syntheses published between 2023 and 2025 consistently emphasize that, despite strong antioxidant effects observed *in vitro* and animal models, well-controlled human intervention studies remain limited, and the clinical relevance of these findings depends on dose, matrix, and bioavailability considerations (45, 88). Differences in algal species, extraction methods, bioactive composition, and experimental conditions may contribute to variability in reported antioxidant efficacy. Consequently, although preclinical findings are largely consistent, further well-designed human studies are required to clarify the clinical relevance and translational potential of these antioxidant properties.

### 6.2 Antibacterial effects

#### 6.2.1 Preclinical evidence (*in vitro* and food model studies)

Brown macroalgae exhibit antibacterial activity against various pathogenic bacteria, including *Staphylococcus aureus*, *Escherichia coli*, *Enterococcus faecalis*, and *Klebsiella pneumoniae*. These effects are largely attributed to the rich phytochemical composition of brown macroalgae, including phenolic compounds, flavonoids, fatty acids, phlorotannins, fucoidan, and alginic acid (89). Most available evidence is derived from *in vitro* assays and food model studies.

A study reported that several brown macroalgal species exerted strong inhibitory effects against *S. aureus*. The members of Phaeophyceae examined included *F. spiralis*, *F. vesiculosus*, *Fucus platycarpus*, *Sargassum vulgare*, *Bifurcaria bifurcata*, *Cystoseira tamariscifolia*, *Cystoseira mediterranea*, *Cystoseira compressa*, *Cystoseira crinita*, *Cystoseira humilis*, and *Cystoseira usneoides* (90). An extract of *S. muticum* demonstrated significant antibacterial activity against a range of human pathogens, including *Salmonella typhi*, *E. coli*, and *S. aureus*, with phenolic and flavonoid compounds contributing to its antimicrobial properties (91). Similarly, a methanol extract of *Padina pavonica* showed strong antibacterial activity against bacterial strains such as *S. aureus* and *Bacillus subtilis*, while also exhibiting antibiofilm and antioxidant activities, highlighting its potential as a multifunctional bioactive agent (92).

In addition to algal extracts, endophytic fungi isolated from brown macroalgae have exhibited promising antibacterial activity, particularly against *E. coli*. The pronounced inhibition zones observed in these studies suggest that algal-associated endophytes may represent potential sources of novel antimicrobial compounds (93). Applications in food systems have also been explored, as *A. nodosum* extract has been shown to extend shelf life by reducing spoilage bacteria and delaying odor formation, supporting its use as a natural food preservative (94).

The antibacterial effects of brown macroalgae are thought to involve multiple mechanisms, including disruption of bacterial cell membranes, inhibition of biofilm formation, and suppression of extracellular enzymatic activity (90, 95–97).

#### 6.2.2 Translational considerations and evidence gaps

Despite consistent antibacterial activity observed in preclinical studies, clinical evidence supporting the use of brown macroalgae-derived compounds as antimicrobial agents remains limited. Antibacterial efficacy varies considerably depending on algal species, extraction method, target bacterial strain, and experimental conditions. Not all algal extracts or isolated compounds demonstrate strong antibacterial activity, underscoring the need for systematic screening and standardization (93, 96).

Moreover, while the presence of diverse bioactive compounds suggests complex and potentially synergistic mechanisms of action, these mechanisms are not yet fully elucidated. Further research is therefore required to clarify structure-activity relationships, evaluate efficacy against antibiotic-resistant bacteria, and assess safety and effectiveness in human-relevant models before clinical or pharmaceutical applications can be reliably considered.

### 6.3 Antidiabetic effects

#### 6.3.1 Preclinical evidence (*in vitro* and animal studies)

Polyphenols derived from brown macroalgae have been shown to reverse metabolic disorders, including hyperglycemia and insulin resistance, in both *in vitro* and *in vivo* studies. The antidiabetic properties of brown macroalgae are primarily linked to their ability to inhibit carbohydrate-hydrolyzing enzymes, increase glucose uptake, and reduce inflammation and oxidative stress, which are crucial in the management of diabetes (98, 99). Promising compounds in the management of diabetes include polyphenols, polysaccharides, phlorotannins, fatty acids, alginate, *ω*-3 PUFAs, fucoidan, phycobiliprotein, phlorotannin, fucoxanthin, and EPA (95). Various studies have reported that these compounds may have potential antidiabetic effects.

In particular, fucoxanthin and fucoidan have been found to have positive effects on glucose metabolism and insulin sensitivity (65, 66). Phlorotannins, a type of polyphenol found in brown macroalgae, have shown potential in reducing insulin resistance through molecular docking studies by binding to PTP1B, a therapeutic target for type 2 diabetes (98). Fatty acids such as 9-hydroxyhexadecanoic acid isolated from brown macroalgae have also demonstrated inhibitory activity against α-amylase and α-glucosidase, enzymes involved in carbohydrate digestion, thereby contributing to postprandial glycemic control (100, 101).

Animal studies provide additional support, although outcomes are not always consistent. For example, studies in diabetic rats reported that some brown macroalgae species, such as *Sargassum glaucescens*, produced slight improvements in indicators such as HbA1c compared to low-dose insulin treatment but did not significantly affect serum glucose or insulin tolerance tests (102). Similarly, *Sargassum boveanum* extracts reduced postprandial blood glucose levels in streptozotocin-induced diabetic mice (103). These findings suggest potential benefits, while also highlighting variability across species and experimental models.

#### 6.3.2 Clinical evidence and translational considerations

Human evidence remains limited but promising. Zaharudin et al. (104) reported that brown macroalgae reduced postprandial glucose and insulin responses in humans exposed to high levels of degradable starch, while also reducing hunger and hyperglycemia and increasing satiety and fullness. In another study, an extract obtained from *Pelvetia babingtonii* exhibited strong α-glucosidase inhibitory activity and effectively suppressed postprandial hyperglycemia (105). Similarly, a phenolic-rich commercial extract derived from *A. nodosum* and *F. vesiculosus* was found to regulate digestive enzymes and carbohydrate absorption, supporting its antidiabetic potential (35).

Additional mechanistic insight has been provided by studies on specific compounds. For example, octaphlorethol A (OPA), a phlorotannin derivative isolated from Ishige foliacea, significantly improved fasting blood glucose levels and glucose tolerance in type 2 diabetic db/db mice by enhancing GLUT4-mediated glucose utilization via AMPK activation in muscle tissue (106, 107). Carotenoids present in alcohol and water extracts of *Sargassum polycystum* have also been reported to lower blood glucose levels and enhance insulin responses (108). Moreover, a meta-analysis of human studies suggested that brown macroalgae consumption may improve postprandial glucose, glycated hemoglobin, and insulin resistance markers, indicating potential benefits for glycemic control in humans (109).

However, the antidiabetic efficacy of brown macroalgae appears to vary considerably depending on algal species, extraction method, dose, and intervention duration. While some species, such as *Sargassum buxifolium*, show strong α-glucosidase inhibitory activity, others demonstrate limited or inconsistent effects (95). Consequently, although preclinical and early clinical findings are encouraging, further well-designed human intervention studies are required to establish optimal formulations, dosing strategies, and long-term safety before brown macroalgae can be reliably integrated into diabetes management strategies.

### 6.4 Anti-inflammatory effects

#### 6.4.1 Preclinical evidence (*in vitro* and animal studies)

Brown macroalgae are increasingly recognized for their potential anti-inflammatory effects, which are attributed to a variety of bioactive compounds including meroterpenoids, fucoidans, phlorotannins, and diterpenoids. These compounds exert anti-inflammatory activity through multiple mechanisms, including modulation of inflammatory signaling pathways, regulation of cytokine production, and attenuation of oxidative stress. Their potential use ranges from dermatological applications to the management of systemic inflammation, highlighting brown macroalgae as promising natural alternatives to conventional anti-inflammatory agents (25). A novel meroterpenoid isolated from *Sargassum macrocarpum* demonstrated significant anti-inflammatory activity, suggesting therapeutic potential (110). Similarly, meroterpenoids derived from *Sargassum siliquastrum* exhibited anti-neuroinflammatory effects by inhibiting the IKK/IκB/NF-κB signaling pathway (111). These compounds have also been reported to reduce oxidative stress, prevent protein denaturation, and modulate immune responses, supporting their potential role in conditions such as atopic dermatitis through targeted regulation of inflammatory pathways (112).

Fucoidan, a sulfated polysaccharide from brown macroalgae, has been extensively studied for its anti-inflammatory and immunomodulatory properties. Preclinical studies indicate that fucoidan inhibits lymphocyte adhesion and diapedesis in the early stages of inflammation and induces apoptosis by suppressing inflammation-related enzymatic activity (28). *In vitro* and animal studies have further shown that fucoidan reduces MAPK phosphorylation, downregulates proinflammatory signaling pathways, increases anti-inflammatory cytokines such as IFN-γ and IL-10, and decreases the expression of TNF-α, IL-1β, and IL-6 (113–115). Consistent with these findings, macroalgae extracts administered to male rats significantly reduced inflammatory markers while enhancing antioxidant defense systems, including SOD, CAT, GSH, GPx, and GST, as well as improving selected hematological parameters (81, 116).

In addition to fucoidan, other brown macroalgal polysaccharides such as alginate and laminarin have shown anti-inflammatory effects in experimental models of nonalcoholic fatty liver disease. Preclinical evidence suggests that these compounds reduce oxidative stress, modulate inflammatory pathways, and beneficially alter gut microbiota composition, thereby contributing to liver protection and improved lipid metabolism (117–119). Studies using macrophage cell lines and animal models have further demonstrated that brown algal extracts can markedly suppress inflammatory responses. For example, low-molecular-weight fucoidan from *Sargassum siliquastrum* reduced proinflammatory cytokine production in lipopolysaccharide-stimulated macrophages, while extracts from *Padina tetrastromatica* inhibited inflammation in adipocytes and mouse models (120, 121).

#### 6.4.2 Translational considerations and evidence gaps

Despite robust anti-inflammatory effects observed in preclinical models, clinical evidence in humans remains limited. While brown macroalgal compounds show promise for applications in skin health, intestinal inflammation, and metabolic liver diseases, challenges related to extraction optimization, formulation, delivery systems (e.g., nanoformulations), and sustainability persist (122). Moreover, although byproducts from species such as *S. fusiforme* have demonstrated anti-inflammatory and gut-modulating effects in animal models, their efficacy and safety in humans have yet to be conclusively established (123).

Therefore, although current *in vitro* and animal studies provide compelling mechanistic insights, well-designed clinical trials are required to confirm efficacy, determine appropriate dosing strategies, and assess long-term safety. Addressing these gaps will be essential for translating the anti-inflammatory potential of brown macroalgae into reliable therapeutic and functional food applications.

### 6.5 Antihypertensive effects

#### 6.5.1 Preclinical evidence (*in vitro* and animal studies)

In the treatment of hypertension, the inhibition of angiotensin-converting enzyme I (ACE I), which converts angiotensin I to angiotensin II, plays a critical role. Angiotensin II is a potent vasoconstrictor that stimulates aldosterone release from the adrenal glands, leading to increased sodium reabsorption and water retention (124). Therefore, inhibition of ACE I reduces aldosterone secretion and contributes to blood pressure regulation (125). Bioactive peptides derived from macroalgal proteins have attracted considerable attention for their potential ACE I inhibitory activity (126). Suetsuna et al. (127) demonstrated that peptides isolated from the brown macroalgal species *U. pinnatifida* exhibited antihypertensive properties. Although brown macroalgae are generally considered less rich in ACE I inhibitory peptides than some other macroalgal groups, preclinical studies suggest that additional bioactive components, including phlorotannins and carbohydrates, may also contribute to ACE I inhibition (126). Brown macroalgae have therefore been identified as promising sources of unique bioactive compounds with potential antihypertensive effects that are not typically found in terrestrial plants.

Several experimental studies have highlighted specific compounds responsible for these effects. For example, *S. macrocarpum* was found to contain meroterpenoids with strong ACE-inhibitory activity, with the chloroform fraction of the algal extract showing potent inhibition (IC₅₀ = 0.18 mg/mL). Molecular docking analyses indicated that these effects result from multiple interactions between meroterpenoids and the ACE active site (128). Other studies have also reported that peptides derived from brown algal proteins exhibit antihypertensive activity in animal models, further supporting their therapeutic potential (129).

*In vivo* evidence has additionally suggested indirect antihypertensive mechanisms. Administration of *S. japonica* to hypertensive rats resulted in significant blood pressure reductions, partly attributed to its alginate content, which enhances fecal sodium excretion. However, these findings also indicate that sodium excretion alone does not fully explain the antihypertensive effects, and that other bioactive components are likely involved (130).

#### 6.5.2 Translational considerations and evidence gaps

Brown macroalgae are rich in polyphenols, particularly phlorotannins, which have been associated with antihypertensive effects through their antioxidant and anti-inflammatory properties and their ability to reduce cardiovascular risk factors (131). Additional compounds, including fucosterol, alginates, and sulfated polysaccharides such as fucoidans, may further contribute to the hypotensive potential of brown macroalgae (132).

However, despite consistent antihypertensive effects observed in *in vitro* and animal studies, clinical evidence in humans remains limited. Variability in algal species, bioactive composition, extraction methods, and bioavailability may influence the magnitude of blood pressure-lowering effects. Moreover, data on long-term safety, optimal dosing, and potential side effects are insufficient. Further well-designed human intervention studies are therefore required to establish the clinical relevance and safety of brown macroalgae-derived compounds for hypertension management.

### 6.6 Antiobesity effects

#### 6.6.1 Preclinical evidence (*in vitro* and animal studies)

There is evidence that brown macroalgae and their bioactive compounds exert anti-obesity effects through multiple mechanisms, including inhibition of adipogenesis, regulation of lipid metabolism, and activation of energy expenditure pathways. Xanthigen, a proprietary mixture of brown macroalgae and pomegranate seed extracts, was shown to exert anti-obesity effects by inhibiting PPARγ expression and activating AMPK phosphorylation (133).

*In vitro* studies have demonstrated that brown algal compounds effectively inhibit adipocyte differentiation. Five bioactive phlorotannins isolated from the edible brown algal species *Ecklonia stolonifera* reduced lipid accumulation in 3 T3-L1 adipocytes, primarily through suppression of C/EBPα and PPARγ expression (134). Among these compounds, phloroglucinol, dioxynodehydroeckol, and eckol exhibited strong inhibitory activity, whereas dieckol, which has a higher molecular weight, showed weaker antiadipogenic effects (134). Similarly, fucosterol isolated from brown macroalgae decreased the expression of adipogenic marker proteins PPARγ and C/EBPα in a concentration-dependent manner (3.125–50 μM) and inhibited adipogenesis in 3 T3-L1 preadipocytes by modulating multiple signaling pathways, including downregulation of SREBP1 and PI3K/Akt- and ERK-dependent FoxO signaling pathways (127, 132).

Additional compounds isolated from brown macroalgae have also shown antiadipogenic activity. Indole derivatives from *Sargassum thunbergii*, particularly 1H-indole-2-carbaldehyde and 1H-indole-6-carbaldehyde, effectively inhibited adipocyte differentiation in 3 T3-L1 cells without cytotoxic effects. These compounds were reported to suppress adipogenesis through activation of the AMPK signaling pathway (135).

Fucoxanthin is another key bioactive compound with well-documented anti-obesity properties. It inhibits lipid accumulation by downregulating PPARγ, C/EBPα, and SREBP1c during adipocyte differentiation, while simultaneously enhancing lipolysis and thermogenesis through stimulation of UCP-1 and β3-adrenergic receptors in white adipose tissue. Moreover, its metabolites, fucoxanthinol and amarouciaxanthin A, have been shown to suppress fat cell differentiation more potently than fucoxanthin itself by inhibiting PPARγ expression (136).

Animal studies further support these findings. Oligosaccharides derived from brown macroalgae improved dyslipidemia, reduced body weight gain, and decreased visceral adiposity in high-fat diet-fed obese rats (136, 137). Sulfated polysaccharides from species such as *Scytosiphon lomentaria* increased energy expenditure and reduced lipid accumulation in zebrafish models through activation of the AMPK pathway (138, 139). Similarly, extracts from *Sargassum subrepandum* significantly reduced body weight and improved liver function in obese rats, while isophloroglucin A from *Ishige okamurae* reduced body weight and fat mass in leptin-deficient mice by modulating leptin signaling pathways (140, 141).

#### 6.6.2 Clinical evidence and translational considerations

Human evidence for the anti-obesity effects of brown macroalgae remains limited but encouraging. Macroalgae supplementation has been reported to significantly reduce body mass index (BMI) and fat mass in overweight and obese adults, particularly when administered for at least 8 weeks. Improvements in lipid profiles, including reductions in total cholesterol and low-density lipoprotein cholesterol, have also been observed (137). A meta-analysis similarly reported significant reductions in BMI and fat mass following macroalgae supplementation, although effects on glucose metabolism were less consistent, with more pronounced benefits observed for lipid-related outcomes (138). Recent evidence syntheses published between 2023 and 2025 indicate that brown macroalgae and their bioactive compounds exhibit antiobesity effects primarily supported by *in vitro* and animal studies, while evidence from human intervention trials remains limited and variable, particularly with respect to dose, formulation, and duration of supplementation (139, 142). Despite these promising findings, variability in individual responses, algal species, bioactive composition, dose, and intervention duration presents challenges for standardization and clinical application. Most available evidence remains preclinical, and data on long-term safety, optimal dosing, and potential interactions with other dietary components or medications are insufficient. Further well-designed human intervention studies are therefore required to confirm efficacy, elucidate underlying mechanisms, and establish standardized guidelines for the use of brown macroalgae-derived compounds in obesity management.

### 6.7 Antihypercholesterolemic effects and impacts on dyslipidemia

#### 6.7.1 Preclinical evidence (*in vitro* and animal studies)

The use of brown macroalgae extracts in dietary supplementation or oral administration has been associated with reductions in total cholesterol, triglycerides, and low-density lipoprotein cholesterol (LDL-C), along with increases in high-density lipoprotein cholesterol (HDL-C). These effects are thought to be mediated by modulation of lipoprotein metabolism, including the regulation of high-affinity receptors involved in cholesterol biosynthesis. In addition, brown macroalgae may increase fecal cholesterol excretion due to the ability of algal compounds to bind dietary cholesterol (132).

Fucoxanthin, a major carotenoid found in brown macroalgae, has been shown in experimental models to reduce hepatic cholesterol and triglyceride levels, suppress cholesterol synthesis through inhibition of HMG-CoA reductase, and increase fecal excretion of cholesterol and triglycerides (39). Fucoxanthin has also been reported to regulate cholesterol absorption by decreasing acyl-CoA: cholesterol acyltransferase (ACAT) mRNA expression while increasing lecithin-cholesterol acyltransferase (LCAT) mRNA levels (39).

Polysaccharides derived from brown macroalgae exert additional hypolipidemic effects. Fucoidan fractions obtained from *A. nodosum* significantly improved lipid profiles in hyperlipidemic mouse models, with effects comparable to atorvastatin, suggesting potential as a natural lipid-lowering agent (143). Fucoidan has also been shown to stimulate reverse cholesterol transport, increase the activity of enzymes involved in serum lipoprotein clearance, and enhance fecal cholesterol excretion (143). Similarly, alginic acid and sodium alginate reduce intestinal cholesterol absorption by binding dietary cholesterol and promoting its elimination through feces (132).

Animal studies further support these findings. For example, *Ecklonia cava* extract from Gijang, Korea, improved serum lipid profiles while reducing body weight gain and fat mass in mice fed a high-fat diet (144). In parallel, other experimental studies have demonstrated reductions in total cholesterol and LDL-C following brown macroalgae supplementation, although effects on HDL-C and triglyceride levels were less consistent (23).

#### 6.7.2 Clinical evidence and translational considerations

Human evidence supporting the antihypercholesterolemic effects of brown macroalgae remains limited but promising. Brown macroalgae are rich in polyphenols and other bioactive compounds with antihyperlipidemic, antihyperglycemic, and anti-inflammatory properties, supporting their potential use in functional foods and dietary supplements (145). Dietary intake of brown macroalgae has been associated with improvements in lipid-related risk factors for cardiovascular disease and metabolic syndrome (146).

However, variability in bioactive composition, extraction methods, and algal origin may substantially influence lipid-lowering efficacy. In particular, structural heterogeneity of fucoidan depending on species and processing conditions can affect biological activity, underscoring the need for standardized extraction and characterization protocols. Moreover, although preclinical findings are robust, more well-designed human intervention studies are required to establish dose–response relationships, long-term safety, and clinical relevance before brown macroalgae can be reliably recommended for dyslipidemia management.

### 6.8 Prebiotic and probiotic effects

#### 6.8.1 Preclinical evidence (*in vitro* and animal studies)

As marine polysaccharides are not absorbed in the gastrointestinal tract and are not digested by gastric or intestinal enzymes, these compounds pass to the lower gastrointestinal region. They are degraded and fermented by the intestinal microbiota, resulting in the production of oligosaccharides, short-chain fatty acids (SCFAs), and other metabolites. SCFAs are rapidly absorbed and provide positive systemic physiological effects for the host. During fermentation, marine polysaccharides and derived oligosaccharides selectively stimulate the growth and activity of specific intestinal microbiota members, such as *Bifidobacterium* spp. and *Lactobacillus* spp., leading to beneficial microbial and ecological changes (6).

Alginate, one of the main structural polysaccharides of brown macroalgae, constitutes approximately 20–29% of the dry weight of *Fucus*, *Ascophyllum*, and *Sargassum* species. This anionic polysaccharide consists of 1,4-linked β-D-mannuronic acid and α-L-guluronic acid units arranged in irregular blocks along the polymer chain. These species also contain fucoidan in lower amounts (10–11% dry weight) (6, 147). Preclinical studies indicate that alginate fermentation increases the abundance of *Bacteroides capillosus* and significantly inhibits intestinal putrefaction, reducing the production of toxic metabolites such as indole, H₂S, and phenol in the large intestine (57).

Fucoidans are soluble homo- or heteropolymeric polysaccharides rich in L-fucose and characterized by irregularly branched, sulfated structures. Due to their high sulfate content, fucoidans cannot be hydrolyzed by human digestive enzymes. Instead, experimental and animal studies demonstrate that fucoidan exerts prebiotic-like effects by selectively modulating gut microbiota composition and increasing *Lactobacillus* spp. abundance in the proximal and distal colon. Similar effects have been reported for galactofucans found in *Laminaria* and *Undaria* species (57, 147). However, preclinical evidence also suggests heterogeneity, as the intestinal effects of fucoidans vary depending on their chemical structure and algal source (6).

Laminarin, a storage β-1,3-glucan containing additional β-1,6 linkages, is present in *Laminaria*, *Ascophyllum*, *Undaria*, and *Fucus* species. This polysaccharide has been shown to be more readily fermentable than alginate, which may explain its greater effectiveness in reducing the production of putrefactive compounds in experimental models (6, 57, 147).

#### 6.8.2 Translational considerations and evidence gaps

Overall, available evidence indicates that brown macroalgae exert consistent prebiotic effects in preclinical models, including shifts in gut microbial composition toward increased *Bacteroidetes* and *Actinobacteria* and reduced *Firmicutes*, as well as enhanced SCFA production—particularly acetate, which is associated with anti-inflammatory and gut health-promoting effects (26, 148). Some brown macroalgae also contain antimicrobial compounds that may further influence microbial ecology and support their use as functional ingredients (95).

However, most findings are derived from *in vitro* fermentation studies and animal models, whereas well-controlled human intervention studies remain limited. Consequently, the magnitude, durability, and clinical relevance of these microbiota-mediated effects in humans are not yet fully established. Further research is therefore required to clarify underlying molecular mechanisms, interindividual variability, and long-term safety before definitive conclusions can be drawn regarding the translational potential of brown macroalgal polysaccharides in human health applications (146).

## 7 Bioavailability, metabolism, and implications for clinical translation

Despite the growing body of evidence supporting the biological activities of brown macroalgae, their clinical translation remains limited, largely due to insufficient data on bioavailability and metabolism. Many bioactive compounds derived from brown macroalgae, including fucoidans, alginates, and phlorotannins, possess high molecular weights and complex polymeric structures that restrict their absorption in the upper gastrointestinal tract. As a result, systemic bioavailability is often low or highly variable, contributing to the scarcity of consistent human evidence and complicating dose standardization across studies (149).

In addition to structural constraints, metabolic transformation and matrix effects further influence the biological fate of macroalgal compounds. Certain carotenoids, such as fucoxanthin, undergo enzymatic conversion to bioactive metabolites (e.g., fucoxanthinol) following ingestion, while polyphenolic compounds may be extensively metabolized by the gut microbiota into smaller phenolic derivatives with distinct bioactivities. The fermentation of indigestible polysaccharides by intestinal microorganisms not only modulates microbiota composition but also generates short-chain fatty acids that may contribute indirectly to host metabolic and anti-inflammatory effects (68, 150). Recent evidence syntheses published between 2023 and 2025 indicate that the clinical translation of brown macroalgal bioactives is primarily constrained by mechanistic factors, including limited intestinal absorption, extensive first-pass metabolism, and biotransformation by gut microbiota, which collectively modulate systemic exposure and downstream biological effects (151). Together, these factors underscore the importance of considering interindividual variability, food matrix interactions, and microbial metabolism when interpreting preclinical findings and designing future clinical trials (45, 147). A clearer understanding of these processes will be essential for improving translational relevance and guiding the development of evidence-based functional foods and nutraceutical applications.

**Figure 1:**
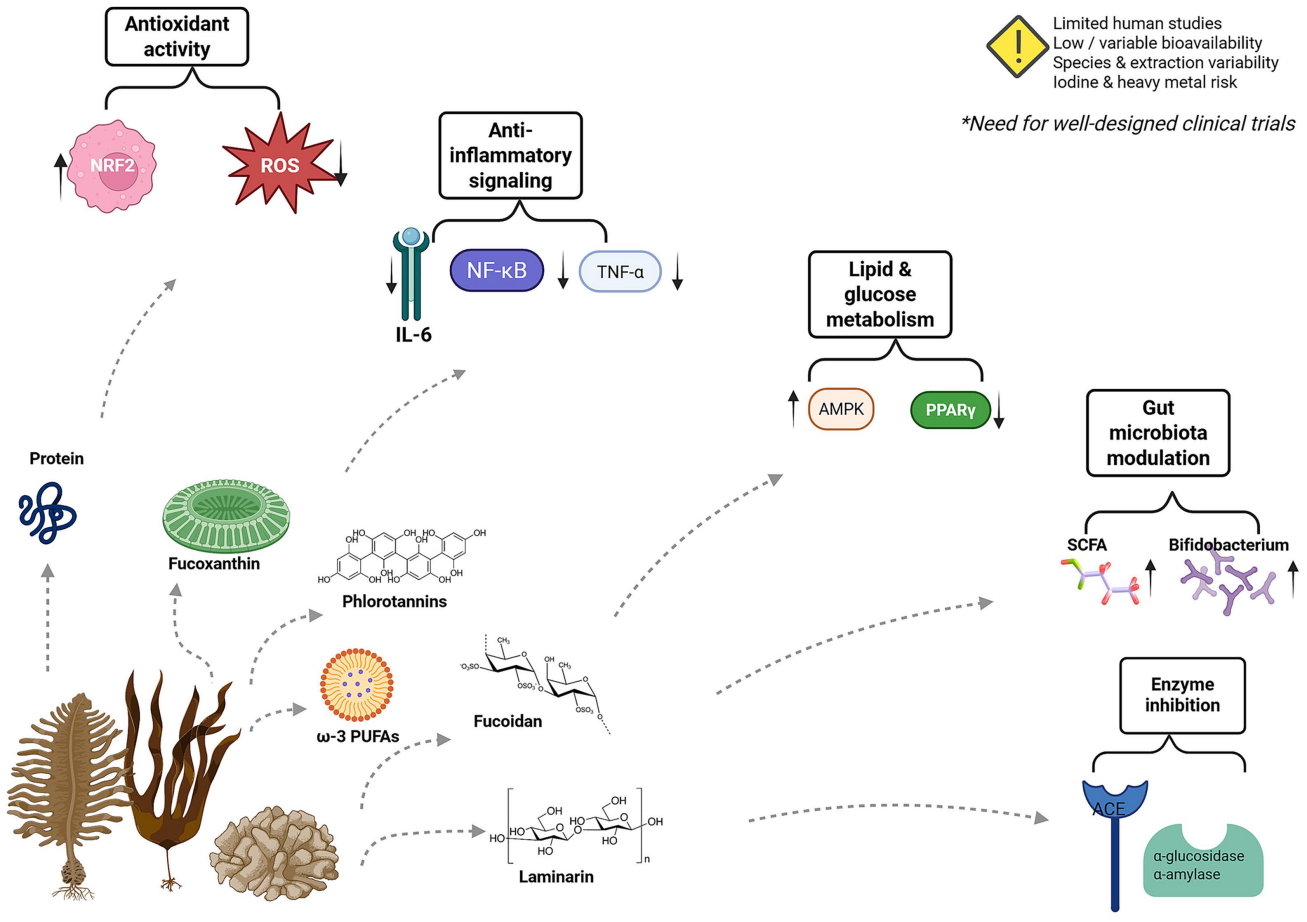
Brown macroalgae and health benefits.

As shown in Table 1, brown macroalgae and their bioactive compounds demonstrate a wide range of health effects supported by both preclinical and clinical studies. In addition the major health benefits associated with brown macroalgae and their bioactive compounds are illustrated in Figure 1.

**Table 1:** Summary of representative preclinical and clinical evidence on the health effects of brown macroalgae and their bioactive compounds.

Health outcome	Algal source/compound	Study type/model	Dose/exposure	Duration	Key findings	Evidence level	References
Antioxidant	Fucoidan isolated from *Hizikia fusiforme*	*In vitro* (Vero cells) and *in vivo* (H₂O₂-stimulated zebrafish)	12.5–50 μg/mL (*in vitro*); pretreatment (*in vivo*)	Acute	Increased cell viability and reduced ROS and apoptosis via Nrf2-mediated upregulation of endogenous antioxidant enzymes; decreased lipid peroxidation and cell death *in vivo*	Preclinical	(79)
	Phenolic-rich extracts of brown macroalgae (e.g., *Alaria*, *Ascophyllum*)	*In vitro*	Micromolar range	Acute	Strong free radical scavenging activity and modulation of oxidative stress-related pathways	*In vitro*	(35, 78)
	Fucosterol (brown algae sterol)	Cellular/animal hepatic models	Variable	Short-term	Reduced ROS production and increased glutathione levels, conferring hepatoprotective effects against oxidative stress	Preclinical	(80, 81)
	Fucoxanthin (various brown macroalgae)	*In vitro* and animal models	Variable	Acute-chronic	Quenching of singlet oxygen, scavenging of free radicals, and inhibition of lipid peroxidation; protection against photo-oxidative damage	Preclinical	(64, 81)
	Supercritical extracts of *Undaria pinnatifida* and *Costaria costata*	*In vitro* (food model: vegetable oil oxidation)	Extracts added to oils (concentration not specified)	Storage period	Reduced lipid oxidation and hydrolysis; extended shelf-life of vegetable oils by ~3 months	*In vitro* (food system)	(82)
Antioxidant/Nephroprotective	Ethanolic and aqueous extracts of *Sargassum binderi*	Animal (cisplatin-induced acute nephrotoxicity)	200 mg/kg (oral)	5 days	Restoration of antioxidant markers (GSH, catalase), reduced lipid peroxidation, and protection against oxidative renal damage	Animal	(85)
Antibacterial	Methanolic extracts of brown macroalgae (e.g., *Cystoseira*, *Dictyota*, *Sargassum*, *Padina* spp.), *Ascophyllum nodosum* extracts, and algae-associated endophytic fungal extracts	*In vitro* (agar diffusion and broth microdilution screening studies) and food model (fish fillet storage assays)	Extracts (disc diffusion; variable concentrations; MIC values typically in the low-mid mg/mL range for spoilage bacteria)	24–48 h (*in vitro*); up to 9 days at 4 °C (food model)	Broad antibacterial activity observed, particularly against Gram (+) bacteria (e.g., *Staphylococcus aureus*, *Enterococcus faecalis*, *Staphylococcus epidermidis*) and selected Gram (−) and antibiotic-resistant strains (e.g., ESBL-producing *Escherichia coli*). In food models, algal extracts inhibited bacterial growth, extended lag phase, reduced growth rate, and significantly delayed spoilage during cold storage	*In vitro*/food model	(90, 91, 93–95)
Antibacterial/Antibiofilm	Methanolic extract of *Padina pavonica*	*In vitro* (antibacterial and biofilm inhibition assays)	Variable concentrations (IC₅₀ ≈ 170 μg/mL for antioxidant activity)	Acute	Strong antibacterial activity against multiple Gram (+) and Gram (−) bacteria and marked inhibition of biofilm formation (88–99%); effects attributed to phenolic- and terpene-rich composition	*In vitro*	(92)
Antidiabetic	Bioactive compounds and extracts from brown macroalgae (e.g., phlorotannins, fatty acids, fucoxanthin, fucoidan; *Sargassum*, *Padina*, *Ascophyllum* spp.)	*In silico* and *in vitro* (α-amylase, α-glucosidase, PTP1B inhibition; molecular docking)	Variable concentrations	Acute	Brown macroalgal compounds inhibited carbohydrate-hydrolyzing enzymes and molecular targets related to insulin resistance, suggesting potential for postprandial glucose regulation	Mechanistic/preclinical	(35, 95, 98–102, 104)
	Brown macroalgae extracts (e.g., *Sargassum boveanum*, *Sargassum glaucescens*)	Animal (STZ- or STZ + HFD-induced diabetic rodents)	Dietary supplementation or oral gavage	Short-medium term	Animal studies reported variable outcomes, ranging from reduced postprandial blood glucose to limited or no effects on fasting glucose, insulin tolerance, or HbA1c, depending on species and dose	Preclinical	(102, 103)
	Edible brown macroalgaes (*Laminaria digitata*, *Undaria pinnatifida*) and brown macroalgae consumption	Human (randomized cross-over trial and meta-analysis)	5 g macroalgaes per meal or dietary intake	Acute to medium term	Human studies indicated reductions in postprandial glucose and insulin responses and improvements in glycaemic markers, with effects	Human	(104, 109)
Anti-inflammatory	Meroterpenoids and diterpenoids from brown macroalgae (e.g., *Sargassum macrocarpum*, *S. siliquastrum*, *Dictyota* spp.)	*In vitro* (LPS-stimulated macrophage and microglial cell models)	μM range (dose-dependent)	Acute	Terpenoid compounds markedly inhibited LPS-induced NO and PGE₂ production and suppressed NF-κB-related inflammatory signaling pathways, indicating strong cellular anti-inflammatory and anti-neuroinflammatory activity	*In vitro* (mechanistic)	(110, 111)
	Fucoidan and other brown algal polysaccharides (alginate, laminarin)	*In vitro* and animal models (macrophages; rodent systemic inflammation and NAFLD models)	Variable doses	Short-chronic	Polysaccharides reduced pro-inflammatory cytokines (TNF-α, IL-1β, IL-6), NO production, and oxidative stress, enhanced antioxidant defenses, and improved liver inflammation in metabolically relevant disease models	Preclinical (animal)	(28, 81, 113–120)
	Brown macroalgae extracts and by-products (e.g., *Padina*, *Sargassum*, *Sargassum fusiforme*)	Animal models (adipose tissue, intestinal, and metabolic inflammation)	Dietary supplementation or extracts	Short-medium term	Algal extracts attenuated inflammatory responses, improved oxidative and metabolic parameters, and supported tissue and intestinal health; however, clinical translation remains limited	Animal	(121–123)
Antihypertensive	ACE-inhibitory peptides derived from brown macroalgae proteins (e.g., *Undaria pinnatifida*)	*In vitro* (ACE inhibition assays) and animal models (spontaneously hypertensive rats, SHR)	Peptide hydrolysates or isolated peptides	Acute-chronic	Low-molecular-weight peptides exhibited strong ACE-inhibitory activity and significantly reduced systolic and diastolic blood pressure in SHR	Preclinical (*in vitro* & animal)	(126–129)
	Meroterpenoids from *Sargassum macrocarpum*	*In vitro* (ACE inhibition) and *in silico* (molecular docking)	Extracts/isolated compounds (IC₅₀ ≈ 0.18 mg/mL)	Acute	Meroterpenoids showed potent ACE inhibition supported by docking analyses indicating favorable interactions with the ACE active site	Mechanistic/preclinical	(128)
	Whole brown macroalgae and polysaccharide-rich preparations (e.g., *Saccharina japonica*, *Saccharina ochotensis*)	Animal models (hypertensive rats: SHR, 2K1C)	Dietary supplementation (≈5%, w/w)	Weeks	Intake of Saccharina species suppressed blood pressure elevation in hypertensive rats; effects were not directly proportional to alginate content, indicating that mechanisms beyond fecal sodium excretion contribute to antihypertensive activity	Animal	(130–132)
Anti-obesity	Polyphenols and indole derivatives from brown macroalgae (e.g., *Sargassum thunbergii*, *Ecklonia* spp.)	*In vitro* (3 T3-L1 preadipocyte differentiation model)	12.5–100 μM	6–8 days	Compounds inhibited adipocyte differentiation and lipid accumulation and downregulated adipogenic transcription factors (PPARγ, C/EBPα, SREBP-1c), partly via AMPK activation	Mechanistic/preclinical	(134, 135)
	Ishophloroglucin A (phlorotannin) from *Ishige okamurae*	Animal (ob/ob leptin-deficient mice; mechanistic cell assays)	2.5 mg/kg/day (oral)	7 weeks	Ishophloroglucin A reduced body weight gain, fat mass, food intake, and serum lipid levels; effects were mediated through activation of hypothalamic leptin signaling pathways (JAK/STAT, ERK, AKT, AMPK)	Animal (preclinical)	(141)
	Edible brown macroalgae and refined or extracted algal components (fucoxanthin, alginates, fucoidans, phlorotannins)	Human (systematic review and meta-analysis of randomized controlled trials)	Dietary supplementation	≥8 weeks	Meta-analysis showed significant reductions in BMI and percentage of fat mass, along with improvements in total and LDL cholesterol; no consistent effects on glucose metabolism were observed	Human (meta-analysis)	(139)
Antihypercholesterolemic/Dyslipidemia	Fucoidan fractions (AFC-1, AFC-2) from *Ascophyllum nodosum*	*In vivo* (high-fat diet-induced hyperlipidemic mice)	Oral administration; fraction-dependent doses (high-dose AFC-2 most effective)	Several weeks (HFD model)	Fucoidan fractions significantly reduced total cholesterol, triglycerides, LDL-C, body weight gain, and hepatic lipid accumulation; effects comparable to atorvastatin; additionally preserved gut microbiota diversity	*In vivo* (animal)	(143)
	Polyphenol-rich extract of *Ecklonia cava*	*In vivo* (HFD-induced obese mice)	300 mg/kg/day oral administration	10 weeks	Reduced serum lipid parameters, body weight gain, fat mass, and hepatic steatosis; downregulated lipogenesis and inflammatory gene expression; increased CYP7A1, indicating enhanced bile acid synthesis	*In vivo* (animal)	(144)
Prebiotic effects	Brown macroalgal polysaccharides (alginate, fucoidan, laminarin)	*In vitro* fermentation studies and animal models	Brown macroalgal polysaccharides resist digestion in the upper gastrointestinal tract and are fermented by gut microbiota, leading to increased SCFA production (particularly acetate), selective stimulation of beneficial bacteria (e.g., *Bifidobacterium*, *Lactobacillus*, *Bacteroides*), and reduced production of putrefactive metabolites	Preclinical	Prebiotic effects	Brown macroalgal polysaccharides (alginate, fucoidan, laminarin)	(6, 26, 57, 147, 148)

^*^This table summarizes representative evidence supporting the reported health effects of brown macroalgae and is not intended to provide an exhaustive overview of all available studies. Screening studies are included as representative *in vitro* evidence and do not imply clinical efficacy. Reported antibacterial and antifungal effects are based primarily on *in vitro* assays and ultrastructural analyses and should not be interpreted as evidence of *in vivo*, field, or clinical effectiveness. References listed are selected examples from the literature and are not intended to comprehensively cover all studies in this area. SHR, spontaneously hypertensive rat; HFD, high fat diet; STZ, streptozotocin.

## 8 Daily intake level and toxicity

Brown macroalgae, as a diverse group of marine organisms, are increasingly recognized for their potential applications in a variety of industries, including food, pharmaceuticals, and aquaculture. However, their use is accompanied by concerns about toxicity and safe consumption levels. The toxic effects of brown macroalgae vary significantly depending on the species and the type of extract used. Some extracts have shown cytotoxic and hemolytic activities, while others are considered safe at certain concentrations. The daily intake level of brown macroalgae is affected by their specific toxicological profiles, which are crucial for determining safe consumption practices (138). From an operational perspective, risk assessment depends primarily on the form of consumption (whole algae, dried powder, extract, or supplement), portion size, and frequency of intake. Occasional culinary use and regular high-dose consumption therefore represent markedly different exposure scenarios (152).

Extracts from brown macroalgae such as *Cystoseira myriophylloides* have shown hemolytic activity against human erythrocytes with varying degrees of cytotoxicity depending on the type and concentration of the extract. Chloroform and butanol extracts have significant hemolytic activities, indicating potential adverse effects when consumed in high doses or for prolonged periods (150). *Cystoseira tamariscifolia* has shown toxic effects in Swiss albino mice, suggesting the need for caution in its use due to potential toxicity at higher concentrations (153). Similarly, a methanolic extract of *Cystoseira trinodis* caused nephrotoxic effects in mice as indicated by histological damage to kidney tissues, and this damage was increased at higher doses (154). These findings indicate that toxic effects are predominantly associated with specific solvent-derived extracts and supraphysiological exposure levels rather than with the consumption of whole macroalgal biomass as food.

Extracts of brown macroalgae such as *Sargassum wightii*, *P. tetrastromatica*, and *Turbinaria conoides* have shown insecticidal potential against *Spodoptera litura*, suggesting that they have bioactive properties that may be toxic to nontarget organisms (155). Although such bioactivities highlight the pharmacological potency of certain compounds, they also emphasize the importance of distinguishing between experimental bioactivity and dietary safety in humans.

Phlorotannin-rich *Sargassum tenerrimum* extracts were evaluated for oral toxicity in rats in a study did not show any adverse effects at doses of up to 800 mg/kg body weight (156). Regular consumption of these algae in reasonable amounts, such as 10 grams per day, can contribute significantly to a balanced diet and help address common mineral deficiencies. Regular consumption scenarios require consideration of cumulative exposure, particularly in individuals consuming brown macroalgae several times per week or using concentrated products such as powders or supplements, as the high iodine content in some algal species may pose health risks if intake is excessive. For example, whole dried kelp products generally pose a higher iodine exposure risk than processed forms, whereas soaking or boiling can reduce iodine content substantially and thereby lower exposure in habitual consumers (157).

While brown macroalgae offer promise for many beneficial applications due to their bioactive compounds, their toxicological profiles necessitate careful consideration of species-specific effects and extraction methods. The presence of potentially toxic elements such as heavy metals further complicates the possibility of their safe use, highlighting the need for comprehensive safety assessments prior to widespread application. In practical terms, regular consumers should preferentially select products sourced from monitored harvesting areas and subject to quality control for heavy metal content. In addition, the antimicrobial properties of certain brown macroalgae species suggest potential benefits in food safety and health, but these must be balanced against their toxic effects (96, 158).

Brown macroalgae are rich in iodine, which is essential for thyroid function. However, excessive iodine intake can lead to thyroid dysfunction, causing conditions such as hyperthyroidism, hypothyroidism, or thyroid autoimmunity. Chronic high iodine consumption can also cause the Wolff–Chaikoff effect, leading to temporary thyroid hormone suppression. Individuals with pre-existing thyroid disease, pregnant or lactating women, children, and those using iodine-containing supplements represent sensitive groups for whom regular macroalgal consumption should be carefully limited or medically supervised. Furthermore, brown macroalgae can accumulate heavy metals such as arsenic, cadmium, lead, and mercury from seawater. Arsenic toxicity is a primary concern as inorganic arsenic is carcinogenic (159). Although most algae contain organic arsenic, long-term high intake can still pose health risks. Therefore, periodic monitoring of thyroid function and exposure markers may be warranted in individuals with habitual intake of brown macroalgae. Alginate, a polysaccharide found in brown macroalgae, may act as a natural laxative, but excessive consumption can cause digestive discomfort, bloating, or diarrhea (160–162).

## 9 Daily intake quantity

Although a specific daily intake has not been established for brown macroalgae, there are recommendations for some species and caution is advised regarding consumption. The recommended daily iodine intake (RDI) for adults is 150 μg. Daily intake levels above 1,100 μg may adversely affect thyroid function. Due to its high iodine content, consumption of kombu (*S. japonica*) should particularly be limited. In addition, daily consumption of 5–10 g of dry wakame (*U. pinnatifida*) is generally considered safe. Due to its high inorganic arsenic content, it is recommended that hijiki (*Sargassum fusiforme*) be avoided or limited (160, 161).

## 10 Limitations

The determination of the components of brown macroalgae, the metabolism of these components, their bioavailability and absorption, algal pharmacokinetics, and the diverse aspects of potential side effects require further research with rigorous clinical studies. Despite the increasing interest in the potential positive effects of brown macroalgae on health, the lack of definitive evidence on their protective health effects and the absence of established dietary consumption habits in some countries limit the generalizability of current findings.

From a methodological perspective, the available literature is dominated by *in vitro* and animal studies, while human intervention studies remain limited in number, often characterized by small sample sizes, short intervention durations, and heterogeneous study designs. In addition, substantial variability in algal species, extraction methods, bioactive composition, and administered doses complicates direct comparison across studies and may influence the consistency of reported outcomes.

More research is needed on the effects of the components of brown macroalgae on disease markers in clinical settings. Therefore, the primary limitation of this review is the very limited number of human studies demonstrating therapeutic effects of brown macroalgae in the treatment and prophylaxis of the diseases and complications discussed here. Moreover, as this work represents a narrative review, no formal quality assessment or risk-of-bias evaluation was performed, which should be considered when interpreting the strength of the presented evidence.

In this context, to make more appropriate generalizations, comprehensive *in vitro*, animal, and well-designed human studies are needed to evaluate the efficacy and safety of brown macroalgae, to clarify their pharmacological and clinical effects, and to identify their mechanisms of action, optimal dosing strategies, and potential risks associated with long-term or high-level consumption.

## 11 Conclusion and future perspective

Brown macroalgae constitute a valuable and sustainable source of bioactive compounds with growing relevance for functional food and nutraceutical development. Key constituents, including fucoidans, phlorotannins, fucoxanthin, and alginates, exhibit diverse biological activities that support metabolic health and may contribute to the prevention of chronic diseases, aligning with broader evidence highlighting the role of plant-based bioactives in clinical and preventive nutrition strategies (162, 163).

However, the effective translation of these bioactivities into clinical and industrial applications remains constrained by several priority knowledge gaps, including the limited number of well-controlled human studies, marked variability in species-specific composition, and the lack of standardized extraction, processing, and dosing protocols. Addressing standardization and safety—particularly with respect to long-term use and iodine exposure—should represent an initial research priority, as these factors directly influence efficacy, reproducibility, and consumer safety.

Future research should further prioritize long-term and population-specific clinical studies, with particular attention to interactions with medications, habitual dietary patterns, and the gut microbiota. Elucidating these factors will be critical for identifying target populations most likely to benefit from brown macroalgal interventions. Integrating bioactive-rich marine resources into evidence-based nutritional frameworks may thereby support both human health and sustainability goals, complementing current plant-forward dietary models emphasized in recent nutrition research (164).

From a translational perspective, future research should systematically address the dose-form-processing relationship of brown macroalgal products. Key variables include the food matrix (whole macroalgae versus extracts or supplements), processing methods such as drying, rehydration, and extraction, and their effects on the stability, bioavailability, and sensory acceptance of bioactive compounds. Processing-related changes may substantially influence iodine content, phenolic integrity, and fucoxanthin stability, thereby altering both efficacy and safety profiles. Integrating these factors within standardized product development frameworks will be critical for the successful conversion of brown macroalgae into safe, effective, and consumer-acceptable functional foods and nutraceuticals.

An additional priority for future research is the management of analytical heterogeneity in the characterization of brown macroalgal bioactive compounds. Variability in extraction conditions and analytical methodologies—such as the use of HPLC or LC-MS for individual compounds versus colorimetric assays for total phenolic content—can lead to substantial differences in reported concentrations and biological interpretations. Similar challenges apply to fucoidan, where molecular weight, degree of sulfation, and reporting units vary widely across studies. Harmonization of analytical protocols, reference standards, and reporting units is therefore essential to improve comparability, reproducibility, and translational relevance of findings related to fucoxanthin, phlorotannins, and fucoidans. The previously published studies investigating the nutrient composition of brown macroalgae are provided in the Supplementary Material (Supplementary Table S1; 165–201).

## Glossary

ACE: Angiotensin-converting enzyme
ACAT: Acyl-CoA: cholesterol acyltransferase
ALT: Alanine aminotransferase
AMPK: Adenosine monophosphate-activated protein kinase
AST: Aspartate aminotransferase
BMI: Body mass index
CAT: Catalase
C/EBPα: CCAAT/Enhancer-binding protein alpha
DHA: Docosahexaenoic acid
EPA: Eicosapentaenoic acid
ERK: Extracellular signal-regulated kinase
GPx: Glutathione peroxidase
GSH: Glutathione
GST: Glutathione S-transferase
HDL-C: High-density lipoprotein cholesterol
HMG-CoA: 3-Hydroxy-3-methylglutaryl-coenzyme A
IC₅₀: Half maximal inhibitory concentration
IL: Interleukin
IKK: IκB kinase
LCAT: Lecithin-cholesterol acyltransferase
LDL-C: Low-density lipoprotein cholesterol
MAPK: Mitogen-activated protein kinase
NF-κB: Nuclear factor kappa B
PPARγ: Peroxisome proliferator-activated receptor gamma
PTP1B: Protein tyrosine phosphatase 1B
PUFA: Polyunsaturated fatty acid
RDI: Recommended daily intake
ROS: Reactive oxygen species
SCFA: Short-chain fatty acid
SOD: Superoxide dismutase
SREBP-1c: Sterol regulatory element-binding protein 1c
TNF-α: Tumor necrosis factor alpha
UCP-1: Uncoupling protein 1
UL: Tolerable upper intake level
