# Supplementary material for: Secret heroes of the sea: brown macroalgae and their bioactive powers—a narrative review
Source: Front Nutr. 2026 Feb 23;13:1766041. doi: 10.3389/fnut.2026.1766041 (PMC12967981; doi:10.3389/fnut.2026.1766041)
Supplement: Supplementary file 1 [file Table_1.docx]

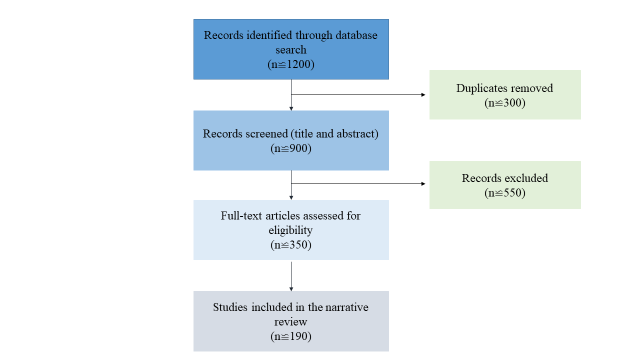


**Supplementary Figure 1.** Literature search and study selection process for this narrative revie

**Supplementary Table 1.** Macronutrients and Fiber Amounts of Brown Macroalgae (g/100g-%)

| **Reference** | **Brown Macroalgae** | **Country** | **Carbohydrate** | | **Protein** | **Lipid** | **Fiber** |
| --- | --- | --- | --- | --- | --- | --- | --- |
| Souto-Prieto et al. (165) | *Laminaria digitata* | Sweden | 69.07±0.13 | 7.75±0.26 | | 1.95±0.11 | - |
|  | *Saccharina latissima* |  | 60.68±0.07 | 9.17±0.02 | | 1.39±0.11 | - |
| Zhu et al. (166) | *Alaria esculenta* | Ireland | - | 17.19±1.33* | | - | - |
| Chin et al. (167) | *Padina australis* | Malaysia | 4.80±1.11 | 3.06 ±0.09 | | 1.82±0.21 | 71.02±2.74 |
|  | *Sargassum binderi* |  | 8.50±0.36 | 7.03±0.04 | | 0.46±0.28 | 72.09±2.98 |
|  | *Sargassum polycystum* |  | 5.07±0.67 | 7.64±0.04 | | 0.63±0.33 | 60.76±1.50 |
| Samarasinghe et al. (168) | *Ascophyllum nodosum* | Denmark and Iceland | - | 11.4±0.18 | | 2.14±0.03 | 42.6 |
|  | *Saccharina lastissima* |  | - | 15.2±0.00 | | 0.68±0.03 | 27.8 |
| Jönsson et al. (169) | *Saccharina latissima* | Ireland | - | 8.1±0.1* | | 2.4±0.1* | 7.1±2.8^a^* |
|  | *Alaria esculenta* |  | - | 11.4±0.1* | | 1.7 ± 0.1* | 3.9±1.2^a^* |
| Meng et al. (47) | *Lessonia nigrescens* | Chile | - | 10.42±0.04 | | 0.87±0.02 | 10.54±0.44 |
|  | *Lessonia flavicans* |  | - | 11.09±0.00 | | 0.48±0.02 | 8.53±0.68 |
|  | *Macrocystis pyrifera* |  | - | 10.42±0.03 | | 1.12±0.02 | 13.77±1.32 |
|  | *Undaria pinnatifida* | China | - | 10.42±0.03 | | 2.31±0.05 | 6.14±0.91 |
|  | *Ascophyllum nodosum* | Peru | - | 6.36±0.00 | | 4.72±0.28 | 5.54±1.85 |
|  | *Ascophyllum nodosum* | France | - | 5.16±0.03 | | 4.25±0.37 | 6.70±0.99 |
|  | *Sargassum muticum* | Japan | - | 16.59±0.12 | | 1.31±0.17 | 12.41±1.13 |
|  | *Sargassum muticum* | Chile | - | 10.95±0.13 | | 1.60±0.06 | 8.38±0.67 |
|  | *Laminaria japonica* | China | - | 13.07±0.03 | | 1.77±0.41 | 12.42±0.40 |

**Table 1.** Continued.

| **Reference** | | | **Brown Macroalgae** | **Country** | | | **Carbohydrate** | **Protein** | | | | **Lipid** | | | **Fiber** |
| --- | --- | --- | --- | --- | --- | --- | --- | --- | --- | --- | --- | --- | --- | --- | --- |
| Oucif et al. (170) | | | *Cystoseira stricta* | West Algerian Coast | | | 354.5±0.4 | 141.4±0.3 | | | | 27.1±0.1 | | | - |
|  |  |  | *Cystoseira compressa* |  |  |  | 396.2±0.7 | 89.1±8.4 | | | | 18.3±1.8 | | | - |
| Olsson et al. (171) | | | *Ascophyllum nodosum* | Sweden | | | 317±17 | 59 | | | | - | | | - |
|  |  |  | *Chorda filum* |  |  |  | 292±33 | 63 | | | | - | | | - |
|  |  |  | *Desmarestia aculeata* |  |  |  | 301±10 | 115 | | | | - | | | - |
|  |  |  | *Fucus serratus* |  |  |  | 287±21 | 71 | | | | - | | | - |
|  |  |  | *Fucus vesiculosus* |  |  |  | 266±25 | 71 | | | | - | | | - |
|  |  |  | *Halidrys siliquosa* |  |  |  | 237±67 | 79 | | | | - | | | - |
|  |  |  | *Laminaria digitata* |  |  |  | 519±42 | 66 | | | | - | | | - |
|  |  |  | *Saccharina latissima* |  |  |  | 557±14 | 69 | | | | - | | | - |
|  |  |  | *Sphacelaria cirrosa* |  |  |  | 267±11 | 120 | | | | - | | | - |
| Nazarudin et al. (172) | | | *Sargassum polycystum* | Malaysia | | | 36.55±1.09* | 8.65±1.06* | | | | 3.42±0.01* | | | 2.75±0.58* |
| Fouda et al. (173) | | | *Cystosira myrica* | Red Sea, Hurghada coast | | | 33.75* | 2.81* | | | | 0.11* | | | - |
|  |  |  | *Padina gymnospora* |  |  |  | 41.66* | 4.13* | | | | 0.13* | | | - |
|  |  |  | *Sargassum aspirofolium* |  |  |  | 39.25* | 3.50* | | | | 0.17* | | | - |
|  |  |  | *Sargassum latifolium* |  |  |  | 41.42* | 4.38* | | | | 0.27* | | | - |
|  |  |  | *Sargassum muticum* |  |  |  | 24.79* | 5.31* | | | | 0.12* | | | - |
|  |  |  | *Turbinaria sp.* |  |  |  | 26.85* | 3.88* | | | | 0.20* | | | - |
| Vilcanqui et al. (133) | | | *Macrocystis pyrifera* | Peru | | | 43.29* | 5.86* | | | | 0.74* | | | - |
| Pirian et al. (174) | | | *Sargassum boveanum* | Persian Gulf | | | - | 21.33±0.35* | | | | 2.02±0.05* | | | - |
|  |  |  | *Sirophysalis trinodis* |  |  |  | - | 14.64±0.20* | | | | 1.27±0.07* | | | - |
| D’armas et al. (175) | | | *Padina pavonika* | Equator | | | 43.39±0.16* | 5.53±0.01* | | | | 0.83±0.01* | | | 4.94±0.16* |
|  |  |  | *Spatoglossum scroederi* |  |  |  | 40.04±0.21* | 5.21±0.02* | | | | 3.07±0.02* | | | 4.28±0.20* |
| Dewinta et al. (176) | | | *Sargassum crassifolium* | Pane Island, Indonesia | | | 3.79* | 6.21* | | | | 0.30* | | | 24.54* |
|  |  |  | *Sargassum cristaefolium* |  |  |  | 7.25* | 8.54* | | | | 0.25* | | | 22.09* |
| Saygılı et al. (177) | | *Padina pavonika* | | Antalya, Türkiye | | | - | 4.29±0.13* | | | 0.67±0.11* | | - | | |
| Uslu et al. (178) | | *Stypopodium schimperi* | | İskenderun, Türkiye | | | - | 8.53±0.11* | | | 6.36±0.22* | | - | | |
|  |  | *Halopteris scoparia* | |  |  |  | - | 9.79±0.02* | | | 2.85±0.3* | | - | | |
|  |  | *Cystoseira compressa* | |  |  |  | - | 9.50±0.3* | | | 2.00±0.5* | | - | | |
|  |  | *Padina pavonika* | |  |  |  | - | 4.20±0.16* | | | 3.18±0.8* | | - | | |
|  |  | *Sargassum vulgare* | |  |  |  | - | 6.29±0.12* | | | 2.58±0.4* | | - | | |
| Bogolitsyn et al. (179) | | *Ascophyllum nodosum* | | Coastal area of ​​the Solovetsky Islands | | | - | 6.60±0.33* | | | - | | - | | |
|  |  | *Fucus vesiculosus* | |  |  |  | - | 6.60±0.34* | | | - | | - | | |
|  |  | *Laminaria digitata* | |  |  |  | - | 7.60±0.38* | | | - | | - | | |
|  |  | *Laminaria saccharina* | |  |  |  | - | 8.80±0.44* | | | - | | - | | |
|  |  | *Ecklonia cava* | | Jeju Island, Republic of Korea | | | - | 9.60±0.54* | | | - | | - | | |
|  |  | *Undaria pinnatifida* | |  |  |  | - | 19.50±1.08* | | | - | | - | | |
| Mandalka et al. (180) | | *Dictyopteris jolyana* | | Brasil | | | 81.0±0.7 | 12.5±0.2 | | | - | | 65.8±1.1 | | |
|  |  | *Spatoglossum schroederi* | |  |  |  | 59.1±0.2 | 21.5±0.2 | | | - | | 59.2±0.4 | | |
|  |  | *Zonaria tournefortii* | |  |  |  | 68.7±0.5 | 10.9±0.4 | | | - | | 54.5±2.3 | | |
| Panjaitan et al. (181) | | *Sargassum sp.* | | Indonesia | | | - | 8.98±0.42* | | | - | | - | | |
| Rosioru (182) | | *Gongolaria barbata* | | Romania | | | 61.95±1.06* | 18.13±2.11* | | | 1.63±0.54* | | 61.07±1.66* | | |
| Jagtap et al. (183) | | *Padina tetrastromatica* | | India | | | - | - | | | - | | 81.4±0.5* | | |
|  |  | *Spatoglossum asperum* | |  |  |  | - | - | | | - | | 76.0±0.5* | | |
|  |  | *Sargassum cinereum* | |  |  |  | - | - | | | - | | 66.3±1.0* | | |
|  |  | *Stoechospermum polypodioides* | |  |  |  | - | - | | | - | | 47.3±6.1* | | |
|  |  | *Sphacelaria rigidula* | |  |  |  | - | - | | | - | | 66.0±6.1* | | |
| Burgos-Díaz et al. (184) | | *Durvillaea incurvata* | | Chile | | | 2.10±0.32 | 8.30±0.41 | | | 0.35±0.27 | | 56.90±2.97 | | |
| Vaghela et al. (185) | | *Sargassum wightii* | | India | | | 3.95±0.13 | 3.51±0.02 | | | 1.8±0.23 | | - | | |
| Yang et al. (186) | | *Sargassum thunbergii* | | China | | | 37.00±1.64* | 7.14±0.41* | | | 7.88±0.88* | | - | | |
| Ullah et al. (187) | | *Padina tetrastromatica* | | Pakistan | | | 49.39±1.62* | 8.30±0.37* | | | 3.75±0.08* | | - | | |
| Alloyarova et al. (34) | | *Himanthalia elongata* | | Pacific and Arctic Oceans | | | 15.00±2.56 | 7.50±1.43 | | | 1.00±0.20 | | 36.00±3.71 | | |
|  |  | *Asperococcus ensiformis* | |  |  |  | 6.45±0.50 | 2.90±0.04 | | | - | | 58.81±2.91 | | |
|  |  | *Ascophyllum nodosum* | |  |  |  | - | 11.40±0.18 | | | 3.00 | | 34.50±2.37 | | |
|  |  | *Saccharina latissima* | |  |  |  | - | 15.20±0.00 | | | 1.50 | | 21.70±3.04 | | |
|  |  | *Lessonia nigrescens* | |  |  |  | - | 10.42±0.04 | | | 0.87±0.02 | | 10.54±0.44 | | |
|  |  | *Laminaria ochroleuca* | |  |  |  | 17.61±0.96 | 6.26±0.09 | | | - | | - | | |
|  |  | *Carpophyllum flexuousm* | |  |  |  | - | 5.90±0.10 | | | 2.60±0.10 | | - | | |
|  |  | *Carpophyllum plumosum* | |  |  |  | - | 7.20±0.20 | | | 1.70±0.00 | | - | | |
|  |  | *Ecklonia radiata* | |  |  |  | - | 7.60±0.00 | | | 3.60±0.20 | | - | | |
|  |  | *Undaria pinnatifida* | |  |  |  | - | 12.50±0.50 | | | 2.20±0.00 | | - | | |
| Winarni et al. (188) | | *Sargassum sp* | | Indonesia | | |  | | 3.04* | | 0.61 | |  | | |
| Dragan et al. (189) | | *Cystoseira barbata* | | Black Sea coast | | | 59.9±1.06* | | 17.9±2.11* | | 1.65±0.54* | | 59.87±1.66* | | |
| Mengisteab et al. (190) | | *Padina boergesenii* | | Eritrea | | | - | | 10.62±0.10* | | - | | 9.19±0.35* | | |
| Mohd Fauziee et al. (191) | | *Sargassum polycystum* | | Malaysia | | | 70.30±1.21* | | 5.51±1.83* | | 0.95±0.28* | | - | | |
|  |  | *Turbinaria ornata* | |  |  |  | 71.65±2.18* | | 4.85±1.37* | | 1.41±0.15* | | - | | |
|  |  | *Padina boryana* | |  |  |  | 74.78±1.63* | | 7.48±0.85* | | 0.73±0.06* | | - | | |
| Ardiana et al. (192) | *Sargassum crassifolium* | | | Indonesia | | 4.03* | | 2.71* | | 1.02* | | | | - | |
| Perumal et al. (193) | *Sargassum polycystum* | | | India | | 25.0* | | 14.8* | | 7.6* | | | | 21.3* | |
| Chowdhury et al. (194) | *Padina pavonika* | | | Bangladesh | | 24.72±6.54* | | 7.64±1.29* | | 0.23±0.08* | | | | 0.83±0.02* | |
|  | *Colpomenia sinuosa* | | |  |  | 17.04±2.44* | | 7.15±0.52* | | 0.20±0.03* | | | | 5.68±0.29* | |
|  | *Sargassum sp.* | | |  |  | 38.75±1.01* | | 13.36±0.15* | | 0.39±0.07* | | | | 7.78±0.42* | |
|  | *Hydroclathrus clathratus* | | |  |  | 10.70±0.16* | | 7.20±0.31* | | 0.15±0.06* | | | | 2.19±0.23* | |
|  | *Dictyota ciliolata* | | |  |  | 14.74±3.31* | | 7.69±1.02* | | 1.68±0.06* | | | | 1.76±0.06* | |
|  | *Petalonia* *fascia* | | |  |  | 43.63±2.72* | | 7.24±0.45* | | 2.75±0.14* | | | | 10.08±0.07* | |
| Choudhary et al. (195) | *Lyengaria stellata* | | | India | | - | | 5±1* | | 7.0±0.5* | | | | 9.0±0.2 * | |
|  | *Lobophora variegate* | | |  |  | - | | 3±0.4* | | 1.0±0.3* | | | | 18.0±0.5* | |
|  | *Padina boergesenii* | | |  |  | - | | 6±1* | | 5.0±0.6* | | | | 11.0±0.5* | |
|  | *Sargassum linearifolium* | | |  |  | - | | 7±1* | | 1.0±0.3* | | | | 12.0±0.5* | |
|  | *Spatoglossum asperum* | | |  |  | - | | 5±1* | | 7.0±0.8* | | | | 13.0±0.8* | |
|  | *Stoechospermum marginatum* | | |  |  | - | | 10±1 * | | 2.0±0.3* | | | | 13.0±0.7* | |
| Polat and Gür (196) | *Dictyota dichotoma* | | | Gulf of Iskenderun | | - | | 4.42±0.29* | | 3.74±0.01* | | | | - | |
|  | *Padina pavonika* | | |  |  | - | | 4.03±0.11* | | 2.16±0.06* | | | | - | |
|  | *Stypopodium schimperi* | | |  |  | - | | 4.73±0.14* | | 3.60±0.06* | | | | - | |
| Múzquiz de la Garza et al. (197) | *Silvetia compressa* | | | Mexico | | - | | 10.4±0.6* | | 2.93±0.10* | | | | - | |
|  | *Cystoseira osmundacea* | | |  |  | - | | 9.1±0.1* | | 1.08±0.07* | | | | - | |
|  | *Ecklonia arborea* | | |  |  | - | | 11.1±0.4* | | 0.56±0.12* | | | | - | |
|  | *Pterygophora californica* | | |  |  | - | | 9.6±0.9* | | 0.55±0.12* | | | | - | |
|  | *Egregia menziesii* | | |  |  | - | | 11.8±0.5* | | 0.67±0.05* | | | | - | |
| El-Manawy et al. (198) | *Padina boergesenii* | | | Egypt | 24.7±0.1* | | | 5.9±0.25* | | 3.8±0.5* | | | | 36.2±2.7* | |
|  | *Polycladia myrica* | | |  | 30.4±0.2* | | | 7.1±0.25* | | 1.3±0.1* | | | | 34.7±2.2* | |
|  | *Hormophysa cuneiformis* | | |  | 23.4±0.2* | | | 4.8±0.6* | | 0.92±0.02* | | | | 27.6±1.6* | |
|  | *Sargassum aquifolium* | | |  | 29.3±0.15* | | | 5.4±0.2* | | 3.1±0.2* | | | | 33.1±2.4* | |
| Imran et al. (199) | *Lyengaria stellata* | | | Pakistan | - | | | 9.61±0.60* | | 14.54±0.44* | | | | 5.29±0.52* | |
| Ganesan et al. (200) | *Padina gymnospora* | | | India | 28.0 ± 0.12 | | | 12.07±0.78 | | 1.4±0.82 | | | | - | |
| Premarathna et al. (201) | *Padina antillarum* | | | Sri Lanka | - | | | 19.66±0.30* | | 4.25±0.10* | | | | 67.59±0.48* | |
|  | *Sargassum ilicifolium* | | |  | - | | | 28.02±0.68* | | 4.45±0.12* | | | | 51.46±0.53* | |
|  | *Sargassum polycystum* | | |  | - | | | 16.15±0.33* | | 4.50±0.21* | | | | 54.49±0.95* | |
|  | *Turbinaria ornate* | | |  | - | | | 23.54±0.53* | | 3.33±0.08* | | | | 62.04±0.58* | |
|  | *Stoechospermum polypodioides* | | |  | - | | | 8.02±0.26* | | 5.63±0.16* | | | | 68.63±0.61* | |
|  | *Sargassum ilicifolium* | | |  | - | | | 43.87±0.37* | | 2.51±0.04* | | | | 45.32±0.42* | |
|  | *Sargassum ilicifolium* | | |  | - | | | 22.89±0.33* | | 1.54±0.04* | | | | 58.92±0.95* | |
|  | *Padina antillarum* | | |  | - | | | 24.83±0.40* | | 2.35±0.03* | | | | 26.64±0.49* | |
|  | *Sargassum ilicifolium* | | |  | - | | | 30.31±0.58* | | 3.30±0.09* | | | | 55.29±0.59* | |

a: water-soluble fiber; values ​​marked with * are expressed as %.
